# Performing well in financial management and quality of care: evidence from hospital process measures for treatment of cardiovascular disease

**DOI:** 10.1186/s12913-015-0690-x

**Published:** 2015-02-01

**Authors:** Gang Nathan Dong

**Affiliations:** Department of Health Policy and Management, Mailman School of Public Health, Columbia University, 600 W 168th Street, 10032 New York, NY USA

## Abstract

**Background:**

Fiscal constraints faced by U.S. hospitals as a result of the recent economic downturn are leading to business practices that reduce costs and improve financial and operational efficiency in hospitals. There naturally arises the question of how this finance-driven management culture could affect the quality of care. This paper attempts to determine whether the process measures of treatment quality are correlated with hospital financial performance.

**Methods:**

Panel study of hospital care quality and financial condition between 2005 and 2010 for cardiovascular disease treatment at acute care hospitals in the United States. Process measures for condition-specific treatment of heart attack and heart failure and hospital-level financial condition ratios were collected from the CMS databases of Hospital Compare and Cost Reports.

**Results:**

There is a statistically significant relationship between hospital financial performance and quality of care. Hospital profitability, financial leverage, asset liquidity, operating efficiency, and costs appear to be important factors of health care quality. In general, public hospitals provide lower quality care than their nonprofit counterparts, and urban hospitals report better quality score than those located in rural areas. Specifically, the first-difference regression results indicate that the quality of treatment for cardiovascular patients rises in the year following an increase in hospital profitability, financial leverage, and labor costs.

**Conclusions:**

The results suggest that, when a hospital made more profit, had the capacity to finance investment using debt, paid higher wages presumably to attract more skilled nurses, its quality of care would generally improve. While the pursuit of profit induces hospitals to enhance both quantity and quality of services they offer, the lack of financial strength may result in a lower standard of health care services, implying the importance of monitoring the quality of care among those hospitals with poor financial health.

*“Experts say that cardiac care is one of the most lucrative areas of medicine. EMH Regional Medical Center (formerly known as Elyria Memorial Hospital) says it generates nearly half its profit from cardiac services… And some local insurers agree that the Elyria hospital provides high-quality care.”*

*− The New York Times, August 18, 2006* (Excerpt from [1])

## Background

Since the 1920s policymakers have been concerned with growing health care costs and seeking to contain costs by adopting new regulations to control hospital rate, restrict investment, and limit medical procedures [2,3]. Many policymakers began to advocate for market-based healthcare systems, in which hospitals have the freedom to set the quantity and quality of service delivery. However, there is a growing concern that the profit driven motives of hospitals may do more harm than good to patients [4,5], and earlier evidence has shown that a market-based healthcare system sometimes has a deleterious effect on service quality [6]. Interestingly, in recent years some hospitals have been performing well in both financial and quality measures. For instance, in the quote at the beginning of this article, EMH Regional Medical Center, an Elyria, Ohio-based nonprofit hospital, not only profited from the lucrative heart procedures but also provided good health care services to their patients.

For hospitals with other business models, however, it is a different story. The recent economic downturn has certainly placed additional pressure on the fiscal resources of acute care hospitals in the United States and abroad (See [7-9] on this subject). These hospitals have been facing rapidly declining incomes and rising labor costs at the same time, and the statistics from the Medicare Payment Advisory Commission (MedPAC) suggest that the aggregate hospital margin have been between −5% and −7% since 2007 and reached −5.4% in 2012. MedPAC’s report to the Congress further predicts that “under current law, payments are projected to decline in 2015; this decline would result in lower margins for all hospitals, including the relatively efficient providers.” (Excerpt from the Report to the Congress: Medicare Payment Policy (March 2014)). This worsening financial situation has forced hospitals to contain costs and achieve high efficiency. [10] calls for healthcare executives and managers to focus their strategic resources on reducing costs, improving operational efficiency, consolidating supply chain infrastructure, strengthening balance sheet, and avoiding growth strategies. To achieve these goals, hospitals may choose to reduce the number of nurses [11-13], increase patient waiting time [14], postpone investments in new technologies [13], lower the degree of compliance with standards [15], or even reduce the use of medical resources [16]. The extreme choice for hospitals experiencing financial difficulties is to close down the entire medical facility, which will limit access to care for all patients, although existing evidence shows that many hospitals continue to operate in spite of financial distress [13,17]. Instead, these hospitals may be avoiding closure by reducing the quality of their services [15] (For public hospitals, there is a strategic alternative: privatization [18]).

### Hypotheses and related literature

The research question naturally arises as to how exactly the long term uncertainty of revenues and costs faced by the hospitals and the new financial performance-driven strategy undertaken by the management could potentially impact the quality of care received by their patients. From a conceptual perspective, hospitals select a particular level of service quality to provide based on the value patients place on quality and the costs of producing that [19]. Hospitals can generate more profits from the extra service revenue by offering higher quality services when patients’ marginal valuation of quality increases with price. While the pursuit of profit induces hospitals to improve the quality and quantity of services they offer, the lack of financial strength results in a lower standard of health care services. Because profit is the difference between revenues earned and costs incurred from providing services, the key condition is that hospitals are capable of controlling costs and maintaining (or improving) the quality of care [20]. Therefore, the net effect of hospital profitability on care quality can be either positive or negative, depending on the magnitude of each factor. To improve service quality and in turn attract more business, hospitals may need to invest in hospital infrastructure, medical equipment, and information technology. Physicians are more likely to refer patients to high-quality facilities and patients may be attracted to these facilities because hospitals offering great amenities and up-to-date technology are perceived as being committed to quality outcomes [21].

A prerequisite for such large-scale investment in infrastructure and technology is the financial capital structure of the hospital and the ability to raise additional funds. If higher bankruptcy risk causes the hospital to take on less debt to finance capital investments and business operations, its debt-to-assets ratio will be lower [22]. At the same time, nonprofit and public hospitals’ ability to raise capital by issuing tax-exempt bonds (also known as conduit bonds) should encourage the use of debt financing, which raises their debt-to-equity ratios [23]. To add another twist to the complications, the optimal capital structure is limited by asset liquidity: the hospital with more liquid assets (e.g., cash and treasury securities on the balance sheet) can afford a higher optimal level of debt. According to [24], illiquidity is a significant private cost of leverage. Following this line of argument, it is easier for the hospital with better liquidity to raise capital for investing in quality-enhancement related projects.

Of courses, the efforts to improve quality can entail substantial costs. We expect that hospitals will have to take actions that lower the cost of providing services while maintaining their quality. They can reduce the size of the nursing workforce, hold down wage and salary levels, and cut back on charity care provision [25-27], and hence we expect a negative relation between the quality of care and the costs of both labor and uncompensated care. On the other hand, evidence has shown that nurse experience and education have a positive effect on quality of care [28,29]. The greater demand for quality of care would encourage hospitals to hire more experienced and highly trained nurses, and this requires higher wages. Although it is strategically desirable for hospitals to improve workforce quality, doing so incurs significant costs of employee compensation and benefits [30,31]. This implies a positive correlation between quality and labor costs. However, increasing the number of highly skilled workers is quality enhancing only to a certain point after which the effect can be diminishing rapidly [32]. It is possible that employing too much of labor and capital inputs can create slack resources, wasteful capacity, dysfunctional operation and organizational chaos that may eventually lead to lower quality [33,34]. Therefore, it is very important to improve efficiency in hospital operations while expanding workforce, and we expect a positive relation between operational efficiency and quality. We summarize our hypotheses in terms of the expected signs of the effects on quality of care in Table 1.

**Table 1:** **Expected sign of the effects on quality of care**

**Determinant**	**Sign**	**Hypothesis**	**Reference**
Profitability	Positive	Hospitals earn additional profits when patients’ marginal valuation of quality increases with price	Spence [19], Newhouse [21]
Debt level	Positive	Borrowing capacity stemming from the benefits of tax-exempt conduit bonds encourages nonprofit hospitals to raise more debt capital	Valvona and Sloan [23]
	Negative	Risk of bankruptcy (or financial distress) and the associated costs cause hospitals to postpone investment and refrain from borrowing.	Wedig et. al. [22]
Asset liquidity	Positive	Hospitals with more liquid assets are more likely to obtain external financing due to higher probability of repayment.	Shleifer and Vishny [24]
Labor costs	Positive	The greater demand for quality of care encourages hospitals to employ a high quality workforce which incurs significant costs of labor.	Feldstein [30], Chiswick [31]
	Negative	Excessive labor costs in the form of compensation and benefits reduce profits.	Sloan [25], Sloan and Steinwald [26]
Charity care costs	Negative	The optimal level of uncompensated care provision depends on balancing the hospital’s marginal benefits and costs, and an oversupply of charity care could negatively impact profits.	Banks et. al. [27]
Operating efficiency	Positive	The elimination of slack resources, wasteful capacity, dysfunctional operation and organizational chaos may lead to high quality of care.	Blegen et. al. [32], Picone et. al. [33], Valdmanis et. al. [34]

Several existing studies have examined how hospital financial pressures have affected the quality of care. [35] empirically studies the relationship between operating margin and patient safety and finds that declining hospital profitability is negatively associated with patient safety indicators for nursing and surgery but not with mortality rates. [16] reports a reduction in cardiac revascularization for Medicaid patients in California after a Medicaid cost-containment program was implemented in 1983. The evidence in [36] that increases in HMO penetration reduced cardiac procedure rates does suggest that the mechanism to lower healthcare costs, driven by the growth of managed care, has adversely affected quality of care, at least to some extent. To specifically examine the relationship between the financial condition of individual hospitals and their business strategies [15] finds that increasing financial pressures as measured by revenues and cashflow lead to cutbacks in medical equipment investment and reductions in standards compliance. Similarly [37] shows that hospitals with lower cashflow to revenues ratio report higher excess incidents. In a slightly different context [38,39] report a positive association between quality of care and operating profit margin in the nursing home industry. Taken together, prior literature suggests that some aspects of patient care quality may be compromised as hospital financial condition deteriorates.

Hospital financial condition is a multi-facet concept that can be measured along many dimensions: capital structure, cost, profitability, liquidity, and efficiency. The existing studies have only examined a small subset of them (including revenue and profit). They generally lack control variables to adjust for confounding factors that may have affected the hospital characteristics and performance measures, and hence do not help identify what areas of financial and operational management posed the greatest challenges to medical process quality control. In addition, the sample size in this line of research is not very large (e.g., N = 82 in [40]). We contribute to the literature in several ways. First, we collect a comprehensive set of hospital financial accounting information from the cost reports of the Centers for Medicare and Medicaid Services (CMS) and process quality measures for treatment of cardiovascular disease from the Hospital Compare database. Second, we decompose the measure of financial condition into distinct components reflecting capital structure (e.g., financial leverage), cost structure (e.g., labor wage and uncompensated care), profitability (e.g., profit margin), asset liquidity (e.g., current ratio and days cash on hand), and operating efficiency (e.g., asset turnover, days patient accounts receivable, average age of plant) (See [41,42] for a textbook treatment of these financial performance indicators). Third, we conduct regression analysis using pooled OLS and first-differences to examine both the cross-sectional and time-series variations in quality of care for patients receiving specific treatment for cardiovascular disease.

## Methods

### Quality measures of treatment processes

The primary source of treatment quality measures is the Hospital Compare data. Each hospital in the database reports the quality of care provided to patients being treated for four clinical conditions (heart attack, heart failure, pneumonia, and surgical infection prevention), and the present research is focused on the process scores for treatment of cardiovascular diseases including heart attack and heart failure. We list the detail description of treatment measures for both clinical conditions in Table 2.

**Table 2:** **Clinical condition and treatment measure**

**Clinical condition**	**Treatment measure**
Heart attack	Average number of minutes before outpatients with chest pain or possible heart attack got a ECG
	Heart Attack Patients Given ACE Inhibitor or ARB for Left Ventricular Systolic Dysfunction (LVSD)
	Heart Attack Patients Given Aspirin at Arrival
	Heart Attack Patients Given Aspirin at Discharge
	Heart Attack Patients Given Beta Blocker at Arrival
	Heart Attack Patients Given Beta Blocker at Discharge
	Heart Attack Patients Given Thrombolytic (Fibrinolytic) Medication Within 30 Minutes Of Arrival
	Heart Attack Patients Given PCI Within 90 Minutes Of Arrival
	Heart Attack Patients Given Smoking Cessation Advice/Counseling
	Outpatients with chest pain or possible heart attack who got aspirin within 24 hours of arrival
	Outpatients with chest pain or possible heart attack who got drugs to break up blood clots
Heart failure	Patients Given ACE Inhibitor or ARB for Left Ventricular Systolic Dysfunction
	Heart Failure Patients Given Discharge Instructions
	Heart Failure Patients Given Smoking Cessation Advice/Counseling
	Heart Failure Patients Given an Evaluation of Left Ventricular Systolic (LVS) Function
	Heart Failure Patients Given ACE Inhibitor or ARB for Left Ventricular Systolic Dysfunction (LVSD)

According to [43], hospitals could report a null value for any process measure if the number of cases is too small or no data are available for this measure; therefore, we drop observations with missing values from all of our analyses. Using the individual quality scores for clinical conditions of heart attack and heart failure (*Score*_*i,t,j*_), we construct a single composite measure of quality of care (*QualityScore*_*i,t*_) as the intervention-sample size-weighted average value for hospital *i* in year *t* with a total number of *SampleSize*_*i,t,j*_ patients for treatment type *j*, and there are *N* types of different treatments for cardiovascular disease as shown in Table 2:

$$ QualityScor{e}_{i,t}={\displaystyle \sum_{j=1}^N\frac{SampleSiz{e}_{i,t,j}}{{\displaystyle \sum_{k=1}^N SampleSiz{e}_{i,t,k}}}\times Scor{e}_{i,t,j}} $$

The estimation of this composite score (*QualityScore*_*i,t*_) is similar to the Denominator-Based Weights (DWB) approach in [44]. The authors also compare this approach with a Bayesian hierarchical latent variable model (BLVM) and find that hospital quality rankings based on both methods are highly correlated. They conclude that such composite score based on DWB is a reasonable measure of hospital-specific process quality because the choice of score composition method does not make much difference if these scores are used solely for assessing hospital performance and monitoring changes in performance over time. Shwartz et al., 2008 [44] does emphasize, though, that this method fails to take into account the hospital size effect. In the following regression analysis, we will control for overall size measure of hospitals in light of this concern.

### Hospital characteristics and financial conditions

Hospital financial statements are obtained from the CMS cost reports. Several unique features of this data set facilitate the current study. First, every year virtually all hospitals in the United States are required to file a cost report in order to receive reimbursement from the federal government for treating Medicare patients. Second, the sample covers various types of hospitals including not-for-profit, public, and for-profit. Third, the financial information in the Cost Reports is more comprehensive and accurate than the previous ones that use survey data, and it represents the whole hospital industry [45]. The alternative data source is the American Hospital Association’s (AHA) hospital survey database. Schrag et al., 2002 [46] compares selected characteristics for hospitals that filed the cost reports and responded to the AHA surveys, and conclude that the two data sources contain very similar information and the CMS cost reports have higher survey-completion rates and better public availability.

After matching hospitals in the Hospital Compare and the Cost Reports we end up with a sample of 13,273 hospital-years. Table 3 lists the number of hospitals in each state and year. Across all years, California, Florida, New York, and Texas are the top four states in terms the number of hospitals in the sample. It should be noted that we exclude observations with incomplete accounting information and treatment quality scores. For example, Maryland has only one observation in 2010 comparing to 19 observations in 2009. We will introduce state and year fixed-effects with standard errors clustered at the state and year levels to address the heterogeneity in different states and years in the following multivariate regression analysis.

**Table 3:** **Number of hospitals in each state and year**

**State**	**2005**	**2006**	**2007**	**2008**	**2009**	**2010**
AK	8	7	7	8	9	5
AL	62	62	63	69	68	44
AR	44	48	36	37	36	23
AZ	26	31	33	41	40	26
CA	187	184	191	195	200	105
CO	27	29	24	30	29	28
CT	20	20	19	19	19	18
DE	2	1	1	2	2	1
FL	99	99	101	107	107	76
GA	75	83	85	88	85	48
HI	11	9	10	9	11	1
IA	52	52	60	69	69	14
ID	9	8	9	9	10	8
IL	88	82	84	93	93	54
IN	48	45	50	49	56	32
KS	53	59	60	58	54	47
KY	49	50	58	57	57	26
LA	53	53	52	52	53	29
MA	36	37	36	38	39	38
MD	16	14	12	11	9	1
ME	23	19	23	27	30	17
MI	49	47	50	48	49	35
MN	47	50	55	58	61	56
MO	59	63	64	63	64	37
MS	48	45	44	47	48	42
MT	18	21	24	25	21	13
NC	57	57	61	59	59	49
ND	16	15	16	14	17	9
NE	35	42	47	51	49	18
NH	18	18	19	20	19	15
NJ	40	36	34	34	32	27
NM	22	27	27	25	23	12
NV	15	14	16	18	17	10
NY	116	119	123	123	121	118
OH	91	97	92	84	85	70
OK	48	61	68	70	71	37
OR	31	34	33	35	32	23
PA	85	83	82	79	82	5
PR	9	7	20	14	13	11
RI	3	3	4	4	5	4
SC	28	32	29	30	30	25
SD	15	17	21	22	26	8
TN	74	64	74	73	71	34
TX	165	166	168	165	158	118
UT	24	23	21	20	21	16
VA	50	48	54	46	44	33
VT	9	11	10	10	9	9
WA	38	38	42	46	41	33
WI	14	16	21	19	17	14
WV	26	33	33	33	31	18
WY	12	10	9	10	11	5

As [44] points out that the quality measure of treatment processes does not take into account the size effect, we need to control for hospital size, ownership, and location, among other financial characteristics including financial leverage, profitability, asset liquidity, operating efficiency, labor costs, and charity care expenses. The standard measure of business size in the hospital financial management literature is *Total Assets* (e.g., [47]) which is reported on the balance sheet of a hospital’s financial statement. Total assets is a comprehensive measure of hospital size because it includes not only the number of beds but also the medical supplies, equipment and facilities. To avoid the problem of skewed distribution of hospital size and potential outliers that may bias the regression results, we use a natural logarithm transformation of the total assets to normalize its distribution: *Size*_*i*_*=* log(*Total Assets*_*i*_).

Capital structure is how a hospital finances its business operations and capital investments by using different sources of funds. To measure the use of debt in the capital structure of hospital *i*, we compute the ratio of its debt to total assets, also known as financial leverage: *Leverage*_*i*_*= Debt*_*i*_/*Total Assets*_*i*_. The higher the ratio, the more debt a hospital has in its capital structure. For for-profit hospitals, leverage is the degree to which a hospital is taking risk to increase profits by utilizing borrowed money. While the assumptions underlying the borrowing behavior of nonprofit and for-profit hospitals are similar, nonprofit hospitals have no tax liabilities and hence no marginal benefit of borrowing [22], although the cost of borrowing is also lower [23]. Nonetheless, the capital structure of nonprofit hospitals are related to their financial distress position [48] and borrowing capacity for new investment [49,50].

*Total Margin*, profit margin, or net margin is a measure of profitability, how much out of every dollar of revenue a hospital actually keeps in earnings, and it is calculated as net income divided by total revenues. To measure a hospital’s ability to pay its obligations (e.g., debt, payables) using its assets (e.g., cash, inventory, receivables), we construct two variables for asset liquidity. The first one is *Current Ratio*_*i*_*= Current Assets*_*i*_/*Current Liabilities*_*i*_. The second one is *Days Cash On Hand*, representing the number of days of operating expenses that a hospital can pay with its cash. When the *Days Cash On Hand* is low, the hospital needs to cut back its spending or increase its fundraising efforts from public, private and philanthropic sources to enable it to fund operations and services.

We use several variables to proxy for hospital operating efficiency. *Asset Turnover*, or *Sales to Assets* ratio, indicates how efficiently a hospital generates revenue on each dollar of its total assets. It is defined as the sales revenue divided by the total assets. *Days Patients Accounts Receivable* is a measure of the average number of days that a hospital takes to receive payment from the payer (e.g., insurance company, patient, government, etc.) after providing health care services to the patient. A high *Days Patients Accounts Receivable* number suggests a low efficiency because the hospital is providing its services to customers on credit and taking longer to get paid. *Average Age of Plant* represents the approximate age of a hospital’s fixed assets. A large *Average Age of Plant* means the hospital is depreciating or replenishing its assets (e.g., medical equipment, information technology) in a slow pace. Vitaliano et al., 1994 [51] attributes the inefficient operation of health care providers to excessive managerial and supervisory personnel and diseconomies of size. Brown et al., 2003 [52] provides evidence that labor costs in hospitals are a much greater portion of total costs than they are for many other industries. Fisher et al., 2006 [53] suggests that executives see labor more as a cost than a profit-driver. To capture the effect of labor costs on health service quality, we take the total salaries from the hospital’s financial statement and scaled it by its total revenue to create the variable: *Salary to Revenue*. In terms of other non-labor costs [54] finds that hospitals subject to greater fiscal pressure generally reduce their provision of uncompensated care relative to hospitals with better financial condition, and the authors warn that policies and practices that encourage lower costs and greater efficiency may undermine the ability of hospitals to support charity care. We obtain the hospital’s total expenses of uncompensated care from the Worksheet S-10 in the cost report and scale it by its total revenue, and we call this variable *Uncompensated Care Cost to Revenue* ratio.

Other institutional and market factors can also influence hospital decisions about the quality of their services. For example, hospital ownership status and geographic location can affect the cost and the valuation of quality. It is well known that managers in for-profit, public and non-profit hospitals have different incentives for financial management and quality control [55]. Service quality in for-profit hospitals is simply driven by contractual and market pressures, whereas it is more likely for reputation concerns among public hospitals (For example, the recent scandals involving false record-keeping and long wait lists at VA hospitals have dominated the news in 2014. Later in the year, President Obama announced executive actions aimed at improving access to quality VA healthcare). Non-profit hospitals are special because they do not have a profit-maximization objective and they do not receive government funding [56]. To control for this hospital ownership effect, we create two dummy variables: *Public* and *Not-for-profit*. The value of *Public* is one for public hospitals and zero otherwise. Similarly, the value of *Not-for-profit* is one for non-profit hospitals and zero otherwise. Baldwin et al., 2004 [57] documents that patients in rural hospitals are more likely than their counterparts located in urban areas to receive lower quality of care, possibly due to their remoteness from urban centers. In light of this observation, we create a dummy variable *Urban* with a value of one if it is an urban hospital and zero if it is a rural hospital. The detailed definition of each dependent and independent variable can be found in Table 4.

**Table 4:** **Variable definitions**

**Variable name**	**Definition**
Quality Score	Intervention sample size weighted average score based on Shwartz et. al. [44]
Natural log of Total Assets	log (Total Assets)
Financial Leverage	Total Liabilities ÷ Total Assets
Total Margin	Net Income ÷ Revenue
Asset Turnover (Sales to Assets)	Revenue ÷ Total Assets
Current Ratio	Current Assets ÷ Current Liabilities
Days Cash On Hand	(Cash + Cash Equivalents) × 365 ÷ Operating Expenses
Days Patients Accounts Receivable	(Accounts Receivable – Allowances for uncollectible) × 365 ÷ Revenue
Average Age of Plant (Year)	Accumulated Depreciation ÷ Annual Depreciation Expense
Salary to Revenue	Salary Expense ÷ Revenue
Public Hospital	1 for government owned hospitals and 0 otherwise
Not-for-profit Hospital	1 for nonprofit hospitals and 0 otherwise

### Statistical analysis

This paper focuses upon assessing the statistical association between hospital financial health and process quality by conducting pooled cross-sectional OLS regressions that relates process quality measure to various hospital financial characteristics. In the first set of analysis, the regression model takes the following form:

$$ QualityScor{e}_{i,t}=\alpha +\beta {X}_{i,t}+{\varepsilon}_{i,t} $$

The dependent variable is the quality measure described in the previous section for hospital *i* in year *t*, The main predictor variables (*X*_*i,t*_) include the natural log of *Total Assets* (size), *Financial Leverage* (capital structure), *Profit Margin* (profitability), *Asset Turnover* (efficiency), *Current Ratio* (liquidity), *Days Cash On Hand* (liquidity), *Days Patients Accounts Receivable* (efficiency), *Average Age of Plant* (efficiency), *Salary to Revenue* (labor cost), *Uncompensated Care Cost to Revenue* (charity care cost), whether it is a *Public Hospital* or *Not-for-profit Hospital*, and whether it is located in an *Urban* area.

Still, there might be differences across states and time that are not captured by these variables and that affect quality score on the LHS and the independent variables on the RHS simultaneously. This may lead to biased and inconsistent parameter estimates; therefore, we add both state and year fixed-effects to the regression models to address this concern.

Next, we are interested in the time-series effect of hospital financial condition on service quality. For example, what will happen to the quality of treatment for cardiovascular patients in the year following a change in hospital profitability, capital structure, liquidity, efficiency, and costs. To answer this question, we take the first-difference of all variables except hospital size (the natural log of *Total Assets*), ownership (*Public Hospital* and *Not-for-profit Hospital*) and location (*Urban Hospital*), and re-estimate our models with the first-differenced variables:

$$ \Delta QualityScor{e}_{i,t}=\alpha +\beta {Z}_{i,t}+\gamma \Delta {X}_{i,t}+{\varepsilon}_{i,t} $$

where the dependent variable is the change in quality from year *t-1* to year *t* for hospital *i*:

$$ \Delta QualityScor{e}_{i,t}= QualityScor{e}_{i,t}- QualityScor{e}_{i,t-1} $$

The independent variables include those with first-difference (*∆X*_*i,t*_): the changes in *Financial Leverage*, *Profit Margin*, *Asset Turnover*, *Current Ratio*, *Days Cash On Hand*, *Days Patients Accounts Receivable*, *Average Age of Plant*, *Salary to Revenue*, *and Uncompensated Care Cost to Revenue*, and those without first-difference (Z_*i,t*_): the natural log of *Total Assets*, *Public Hospital*, *Not-for-profit Hospital*, and *Urban Hospital*. This first-difference regression method removes both the latent heterogeneity and the time-invariant effects from the model (See [58] for a textbook treatment of this topic, and [59] for excellent discussion of the advantages of first-differencing in regression analysis.). In the context of this paper, for example, if the results from our previous estimations suggest that hospitals with higher leverage, profitability, liquidity or efficiency are associated with better quality score, this could be caused by the time-invariant characteristics of the hospitals, whereas the results from the first-differences estimation will suggest that the change of quality score is related to the changes of leverage, profitability, liquidity or efficiency in the same hospital over time.

## Results

The summary statistics of all variables for the entire sample (N = 13,273) are shown in Section A of Table 5. The average quality score is 0.79 with the minimum being 0.1 and the maximum being close to 1. In our sample, 57% are nonprofit hospitals and 22% are public hospitals. Overall, public hospitals have a lower mean and wider dispersion of quality scores than their nonprofit counterparts. In addition, non-profit hospitals have larger and older assets, higher leverage, higher profitability, and higher efficiency (as measured in asset turnover and days patients accounts receivable), but lower liquidity (as measured in current ratio, and days cash on hand) and labor costs than public hospitals.

**Table 5:** **Summary statistics**

**A. Entire sample (N = 13,273)**					
**Variable**	**N**	**Mean**	**Standard deviation**	**Minimum**	**Maximum**
Quality Score	13,273	0.793	0.186	0.102	0.998
Public	2,871	0.685	0.231	0.102	0.998
Nonprofit	7,587	0.830	0.152	0.102	0.998
Natural log of Total Assets	13,273	17.9	1.39	11.4	22.5
Public	2,871	17.3	1.47	11.4	21.8
Nonprofit	7,587	18.2	1.35	12.2	22.5
Financial Leverage	13,273	0.611	0.483	0.038	2.91
Public	2,871	0.459	0.338	0.038	2.91
Nonprofit	7,587	0.548	0.362	0.038	2.91
Profit Margin	13,273	2.74%	8.78%	−30.8%	25.9%
Public	2,871	2.36%	7.98%	−30.8%	25.9%
Nonprofit	7,587	2.70%	7.04%	−30.8%	25.9%
Asset Turnover (Sales to Assets)	13,273	1.33	0.728	0.372	4.54
Public	2,871	1.19	0.666	0.372	4.54
Nonprofit	7,587	1.25	0.643	0.372	454
Current Ratio	13,273	2.61	2.23	0.145	14.7
Public	2,871	3.33	2.65	0.145	14.7
Nonprofit	7,587	2.51	2.13	0.145	14.7
Days Cash On Hand	13,273	41.0	56.7	0.01	306
Public	2,871	65.2	70.8	0.01	306
Nonprofit	7,587	43.2	53.2	0.01	306
Days Patients Accounts Receivable	13,273	56.5	23.4	11.5	177
Public	2,871	63.8	27.2	11.5	177
Nonprofit	7,587	52.8	19.9	11.5	177
Average Age of Plant (Years)	13,273	14.2	18.9	0.782	157
Public	2,871	15.1	18.2	0.782	157
Nonprofit	7,587	16.3	21.0	0.782	157
Salary to Revenue	13,273	40.5%	8.80%	23.0%	69.1%
Public	2,871	45.1%	9.17%	23.0%	69.1%
Nonprofit	7,587	41.1%	7.48%	23.0%	69.1%
Uncompensated Care Cost to Revenue	9,584	12.5%	9.48%	0.93%	59.1%
Public	1,660	14.9%	11.9%	0.93%	59.1%
Nonprofit	5,575	11.9%	8.63%	0.93%	59.1%
Public Hospital	13,273	21.6%	41.2%	0	1
Not-for-profit Hospital	13,273	57.2%	49.5%	0	1
Urban Hospital	13,174	54.9%	49.7%	0	1
**B. Sub-sample with non-missing values for Urban Hospital and Uncompensated Care Cost (N = 9,570)**					
**Variable**	**N**	**Mean**	**Standard deviation**	**Minimum**	**Maximum**
Quality Score	9,570	0.826	0.155	0.102	0.998
Public	1,655	0.741	0.203	0.102	0.998
Nonprofit	5,566	0.854	0.125	0.102	0.998
Natural log of Total Assets	9,570	18.17	1.28	11.4	22.5
Public	1,655	17.7	1.47	11.4	21.8
Nonprofit	5,566	18.5	1.19	12.2	22.5
Financial Leverage	9,570	0.630	0.497	0.038	2.91
Public	1,655	0.459	0.351	0.038	2.91
Nonprofit	5,566	0.553	0.355	0.038	2.91
Profit Margin	9,570	2.92%	8.91%	−30.8%	25.9%
Public	1,655	2.10%	8.11%	−30.8%	25.9%
Nonprofit	5,566	2.85%	7.40%	−30.8%	25.9%
Asset Turnover (Sales to Assets)	9,570	1.33	0.712	0.371	4.538
Public	1,655	1.18	0.667	0.371	4.538
Nonprofit	5,566	1.24	0.627	0.371	4.538
Current Ratio	9,570	2.53	2.20	0.145	14.796
Public	1,655	3.21	2.57	0.145	14.796
Nonprofit	5,566	2.50	2.19	0.145	14.796
Days Cash On Hand	9,570	37.6	54.5	0.01	306
Public	1,655	61.7	66.5	0.01	306
Nonprofit	5,566	42.8	54.4	0.01	306
Days Patients Accounts Receivable	9,570	54.5	22.3	11.5	177
Public	1,655	62.8	27.2	11.5	177
Nonprofit	5,566	51.7	19.7	11.5	177
Average Age of Plant (Years)	9,570	14.3	19.6	0.782	158
Public	1,655	15.1	18.9	0.782	158
Nonprofit	5,566	16.8	22.2	0.782	158
Salary to Revenue	9,570	39.4%	8.53%	23.0%	69.1%
Public	1,655	44.3%	9.13%	23.0%	69.1%
Nonprofit	5,566	40.5%	7.33%	23.0%	69.1%
Uncompensated Care Cost to Revenue	9,570	12.5%	9.48%	0.93%	59.1%
Public	1,655	15.0%	11.9%	0.93%	59.1%
Nonprofit	5,566	11.9%	8.64%	0.93%	59.1%
Public Hospital	9,570	17.3%	37.8%	0	1
Not-for-profit Hospital	9,570	58.2%	49.3%	0	1
Urban Hospital	9,570	65.0%	47.7%	0	1
**C. Two-sample t-tests for differences in means**					
**Variable**	**Size of entire sample**	**Size of sub-sample**	**Difference**		**t-test**
Quality Score	13,273	9,570	−0.033***		−14.59
Natural log of Total Assets	13,273	9,570	−0.27***		−15.17
Financial Leverage	13,273	9,570	−0.019***		−2.88
Profit Margin	13,273	9,570	−0.0018		−1.52
Asset Turnover (Sales to Assets)	13,273	9,570	0.002		0.21
Current Ratio	13,273	9,570	0.080***		2.70
Days Cash On Hand	13,273	9,570	3.4***		4.57
Days Patients Accounts Receivable	13,273	9,570	2.0***		6.55
Average Age of Plant (Years)	13,273	9,570	−0.10		−0.39
Salary to Revenue	13,273	9,570	0.011***		9.49
Public Hospital	13,273	9,570	0.043***		8.17
Nonprofit Hospital	13,273	9,570	−0.01		−1.51

Difference is shown with ***, ** and * indicating its statistical significant level of 1%, 5% and 10% respectively.

The average hospital size is $59.4 million (corresponding to the natural exponential of 17.9) and the total liabilities of an average firm are about 61.1% of its total assets. The highest financial leverage of 291% suggests that some hospitals in our sample are in severe financial distress, meaning their total liabilities are much larger than total assets. On average, the total profit margin is 2.74% with the most profitable hospital making $25.9 net income out of $100 revenue. Not surprisingly, labor costs constitute a large portion, roughly 40%, of the total revenue. The average current ratio is 2.61, and the average age of hospital assets (plant) is 14.2 years. It takes about 41 days for an average hospital to exhaust all of its cash and 56.5 days to collect its patience service revenue.

The Pearson’s correlations of the entire sample are reported in Section A of Table 6. An examination of the correlation matrix indicates that the correlations between independent variables are generally small. This low correlation among the covariates helps prevent the problem of multicollinearity that causes high standard errors and low significance levels when both variables are included in the same regression. However, the correlation between *Public Hospital* and *Not-for-profit Hospital* (-0.61) is quite high. Although the additional VIF (Variance Inflation Factor) test does not reveal any evidence of multicollinearity, to be cautious, we will separate these two variables in different regression specifications to avoid potential multicollinearity problems.

**Table 6:** **Correlation matrix**

**A. Entire sample (N = 13,273)**												
	**Natural log of total assets**	**Financial leverage**	**Profit margin**	**Asset turnover (sales to assets)**			**Current ratio**	**Days cash on hand**	**Days patients accounts receivable**	**Average age of plant**	**Salary to total revenue**	**Public hospital**
Financial Leverage	−0.13											
Profit Margin	0.21	−0.26										
Asset Turnover (Sales to Assets)	−0.47	0.37	−0.10									
Current Ratio	−0.02	−0.31	0.20	−0.18								
Days Cash On Hand	0.07	−0.23	0.14	−0.29			0.46					
Days Patients Accounts Receivable	−0.13	0.01	−0.12	−0.15			0.09	0.05				
Average Age of Plant	0.05	−0.06	−0.02	−0.02			−0.02	0.03	0.12			
Salary to Revenue	−0.27	−0.05	−0.38	0.04			−0.05	0.08	0.19	0.10		
Public Hospital	−0.22	−0.17	−0.02	−0.10			0.17	0.22	0.17	0.02	0.27	
Not-for-profit Hospital	0.29	−0.15	−0.01	−0.12			−0.05	0.05	−0.16	0.12	0.07	−0.61
**B. Sub-sample with non-missing values for Urban Hospital and Uncompensated Care Cost (N = 9,570)**												
	**Natural log of total assets**	**Financial leverage**	**Profit margin**	**Asset turnover (sales to assets)**	**Current ratio**	**Days cash on hand**	**Days patients accounts receivable**	**Average age of plant**	**Salary to total revenue**	**Uncompensated care cost to revenue**	**Public hospital**	**Not-for-profit hospital**
Financial Leverage	−0.18											
Profit Margin	0.21	−0.23										
Asset Turnover (Sales to Assets)	−0.50	0.38	−0.07									
Current Ratio	0.01	−0.30	0.19	−0.16								
Days Cash On Hand	0.12	−0.22	0.11	−0.28	0.41							
Days Patients Accounts Receivable	−0.09	−0.02	−0.10	−0.16	0.08	0.04						
Average Age of Plant	0.09	−0.08	0.01	−0.04	−0.02	0.04	0.10					
Salary to Revenue	−0.17	−0.07	−0.39	0.00	−0.04	0.11	0.19	0.09				
Uncompensated Care Cost to Revenue	−0.06	0.10	−0.18	0.13	−0.05	−0.02	0.14	0.03	0.36			
Public Hospital	−0.15	−0.16	−0.04	−0.10	0.14	0.20	0.17	0.02	0.26	0.12		
Not-for-profit Hospital	0.31	−0.18	−0.01	−0.15	−0.01	0.11	−0.15	0.15	0.14	−0.07	−0.54	
Urban Hospital	0.46	0.09	0.01	0.05	−0.11	−0.07	−0.11	0.05	−0.12	−0.00	−0.23	0.13

Because not all hospitals report the costs of uncompensated care and the classification of urban or rural hospital location in their cost reports every year, we construct a sub-sample (N = 9,570) that includes these two variables with non-missing values along with other covariates. The summary statistics and correlations are shown in Section B of Tables 5 and 6 respectively. The two-sample t-tests of unequal sample-size and variance for differences in means (Section C of Table 5) reveal that on average the hospitals in the sub-sample have better quality score, larger assets, higher financial leverage, better efficiency (days patients accounts receivable), lower labor costs and asset liquidity (current ratio, days cash on hand) than those in the entire sample.

Table 7 provides the results of the coefficient estimates for the statistical relationship between quality of care and various hospital characteristics and financial conditions. The dependent variable in all specifications is the hospital’s *Quality Score*. The independent variables include hospital size (*Total Assets*), capital structure (*Financial Leverage*), profitability (*Profit Margin*), operating efficiency (*Asset Turnover*, *Days Patients Accounts Receivable*, and *Average Age Of Plant*), asset liquidity (*Current Ratio* and *Days Cash On Hand*), and labor costs (*Salary to Revenue*). In specifications (3) and (4) we add variables that measure the amount of charity care that the hospital provides (*Uncompensated Care Cost to Revenue*) and whether it is located in an urban area (*Urban Hospital*). Unfortunately, not all hospitals report their uncompensated care costs and urban/rural classification in the cost reports in each year, and hence we have to drop observations with missing values for these two variables to construct a subsample of smaller size. Besides, the two hospital ownership variables (*Public Hospital* and *Not-for-profit Hospital*) are highly correlated with each other (−0.61), we run two separate regressions with *Public Hospital* in specification (1) and (3) and *Not-for-profit Hospital* in specification (2) and (4) to avoid multicollinearity.

**Table 7:** **Regression of quality score on hospital financial characteristics**

**Dependent variable: quality Score**	**(1)**	**(2)**	**(3)**	**(4)**
Natural log of Total Assets	0.0720*** (58.44)	0.0724*** (57.67)	0.0565*** (38.29)	0.0554*** (36.74)
Financial Leverage	0.00189 (0.622)	0.00830*** (2.703)	0.0105*** (3.543)	0.0154*** (5.155)
Profit Margin	−0.0258 (−1.530)	−0.0318* (−1.877)	−0.0238 (−1.417)	−0.0257 (−1.524)
Asset Turnover (Sales to Assets)	0.0345*** (14.86)	0.0371*** (15.94)	0.0193*** (7.827)	0.0200*** (8.073)
Current Ratio	0.000758 (1.145)	0.000718 (1.079)	−0.000288 (−0.435)	−0.000326 (−0.490)
Days Cash On Hand	−0.000139*** (−5.384)	−0.000199*** (−7.743)	−0.000117*** (−4.405)	−0.000166*** (−6.293)
Days Patients Accounts Receivable	−0.000116** (−1.974)	−0.000101* (−1.705)	−0.000159** (−2.568)	−0.000145** (−2.332)
Average Age of Plant	2.93e-05 (0.444)	−3.26e-05 (−0.487)	0.000121* (1.870)	6.75e-05 (1.031)
Salary to Revenue	−0.137*** (−7.375)	−0.212*** (−11.77)	−0.0890*** (−4.360)	−0.153*** (−7.674)
Uncompensated Care Cost to Revenue			−0.123*** (−8.112)	−0.121*** (−7.911)
Public Hospital	−0.0529*** (−15.05)		−0.0416*** (−10.92)	
Not-for-profit Hospital		0.0248*** (8.371)		0.0194*** (6.384)
Urban Hospital			0.0179*** (5.442)	0.0209*** (6.337)
Constant	−0.527*** (−15.14)	−0.534*** (−15.24)	−0.231*** (−6.298)	−0.211*** (−5.687)
Year Fixed-effects	Yes	Yes	Yes	Yes
State Fixed-effects	Yes	Yes	Yes	Yes
N	13,273	13,273	9,570	9,570
Adj. R-squared	0.430	0.424	0.410	0.406

The dependent variable is quality score. The independent variables include the natural log of total assets, financial leverage, profit margin, asset turnover (sales to asset), current ratio, days cash on hand, days patient accounts receivable, average age of plant, total salary to revenue, and three dummy variables: public hospital, nonprofit hospital, and urban hospital. All specifications use OLS regressions with year and state fixed-effects. z-statistics are shown in the parentheses with ***, ** and * indicating its statistical significant level of 1%, 5% and 10% respectively.

The results suggest that nonprofit hospitals provide better quality patient care than public hospitals. Across all specifications, hospitals with larger size (*Total Assets*), more use of debt in capital structure (*Financial Leverage*), and better operating efficiency (higher *Asset Turnover* and fewer *Days Patients Accounts Receivable*) are associated with better quality of care, whereas those with better asset liquidity (more *Days Cash On Hand*), and higher costs (*Salary to Revenue* and *Uncompensated Care Cost*) are associated with lower service quality. The finding of the negative effect of uncompensated care costs on quality is not surprising either. In general, a higher spending on uncompensated care will reduce profit, and hence the quality of care. On average, a hospital with its asset size one-standard deviation above the mean has a quality score 9.9% above the sample mean. The average effects of financial leverage, asset turnover, days patients accounts receivable, days cash on hand, wage cost, and uncompensated care cost on quality score are 0.9%, 1.8%, −0.5%, −1.1%, −1.7%, and −1.5% respectively. It is noted that the coefficient estimate of financial leverage is not statistically significant at the 1% or 5% level in specification (1); however it becomes significant at the 1% level in specifications (2) to (4). It is also noted that the statistical relationships exhibited in the subsample (specifications 3 and 4) do not differ significantly from those in the entire sample (specifications 1 and 2), even though on average, hospitals in this subsample have better quality score, larger assets, higher financial leverage, better efficiency, lower labor costs and asset liquidity than those in the entire sample (Section C of Table 5). In addition, the positive effect of being located in urban areas on the quality of care is consistent with the evidence in [57].

The finding that financial leverage, operating efficiency, asset liquidity, and costs are important contributing factors to quality of care could be caused by the time-invariant (and omitted) characteristics of the hospitals. Therefore, it may not have the statistical power to answer the question: what will happen to the service quality of patient care when hospital financial performance improves over time? As we discussed in the methodology section, the first-difference method can be used to address the omitted variable problem by removing both the latent heterogeneity and the time-invariant effects from the model. We re-estimate our regression models using “first-differences” of data: ∆*Y*_*i,t*_=*α*+*β*∆*X*_*i,t*_+*ε*_*i,t*_ and report the coefficient estimates in Table 8.

**Table 8:** **Regression of changes in quality score on changes in hospital financial performance**

**Dependent variable: quality score**	**(1)**	**(2)**	**(3)**	**(4)**
Natural log of Total Assets	−0.00101 (−1.198)	−0.000460 (−0.538)	−0.000755 (−0.773)	−5.59e-05 (−0.0558)
∆Financial Leverage	0.0150** (2.248)	0.0148** (2.227)	0.0131** (1.979)	0.0126* (1.903)
∆Profit Margin	0.0505*** (3.022)	0.0499*** (2.988)	0.0316* (1.841)	0.0307* (1.787)
∆Asset Turnover (Sales to Assets)	0.000745 (0.195)	0.00117 (0.306)	−0.00472 (−1.183)	−0.00463 (−1.160)
∆Current Ratio	−0.000143 (−0.180)	−0.000179 (−0.224)	0.000119 (0.145)	9.84e-05 (0.119)
∆Days Cash On Hand	2.22e-05 (0.492)	2.47e-05 (0.548)	3.54e-05 (0.724)	3.86e-05 (0.789)
∆Days Patients Accounts Receivable	−4.25e-05 (−0.629)	−3.61e-05 (−0.534)	1.09e-08 (0.000149)	5.95e-06 (0.0813)
∆Average Age of Plant	−0.000136 (−1.085)	−0.000129 (−1.025)	1.21e-05 (0.103)	1.86e-05 (0.158)
∆Salary to Revenue	0.0802** (2.337)	0.0772** (2.252)	0.0515 (1.366)	0.0460 (1.221)
∆Uncompensated Care Cost to Revenue			−0.0326 (−1.198)	−0.0329 (−1.205)
Public Hospital	−0.00535* (−1.917)		−0.00983*** (−3.239)	
Not-for-profit Hospital		−0.00362 (−1.466)		−0.00733*** (−2.952)
Urban Hospital			−6.65e-05 (−0.0249)	0.00155 (0.588)
Constant	0.0552** (2.250)	0.0460* (1.880)	0.0504** (2.049)	0.0382 (1.545)
Year Fixed-effects	Yes	Yes	Yes	Yes
State Fixed-effects	Yes	Yes	Yes	Yes
N	9,782	9,782	6,719	6,719
Adj. R-squared	0.079	0.079	0.128	0.128

The dependent variable is the change of quality score. The independent variables include the natural log of total assets, changes in financial characteristics (financial leverage, profit margin, asset turnover, current ratio, days cash on hand, days patient accounts receivable, average age of plant, and total salary to revenue), and three dummy variables: public hospital, nonprofit hospital, and urban hospital. All specifications use OLS regressions with year and state fixed-effects. z-statistics are shown in the parentheses with ***, ** and * indicating its statistical significant level of 1%, 5% and 10% respectively.

The results show that the changes in patient care quality are positively related to the changes in financial leverage, profitability and labor costs of the same hospital over time. On average, in the year following a 1% increase in the rate of changes in *Profit Margin*, the rate of changes in *Quality Score* will rise by 0.42%. The similar changes in *Financial Leverage* and *Salary to Revenue* will improve *Quality Score* by 1.17% and 0.36% respectively. The coefficient estimates of the changes in *Asset Turnover*, *Days Patients Accounts Receivable*, and *Days Cash On Hand* are no longer statistical significant. The lack of significance of the coefficients on the efficiency and liquidity measures suggests that the heterogeneity in the quality of care may be driven by cross-sectional variations rather than time-series changes in hospital operating efficiency and asset liquidity.

## Discussion

Since the 1970s we have been attempting to reform our financing of health care and moving towards deregulation and a more market-based health care system. From the standpoint of hospitals operating in this market, there are three important aspects of a market-based system: 1) relatively unrestrained pursuit of profit, 2) easy access to capital, and 3) providing extensive choices of health care services [60]. Some hospitals, including the EMH Regional Medical Center in the quote at the beginning of this article, not only made money from their business model but also provided good quality patient care. However, the recent economic downturn has certainly not only affected the health of some subgroups of the population but also placed additional pressure on the fiscal resources of acute care hospitals. Many hospitals have been facing declining incomes, and this situation has forced hospitals to cut costs and improve financial and operational efficiency. There naturally arises the research question of how this new financial performance-driven strategy could potentially impact the quality of care received by patients. Unfortunately, prior studies on this issue generally lack control variables to adjust for confounding factors that may have affected hospital characteristics, financial performance, and service quality. We add to the literature by constructing a comprehensive set of variables that measure hospital size, financial leverage, asset liquidity, operating efficiency, profitability, labor costs, and charity care costs from the CMS cost reports and quality measures for cardiovascular disease treatment from the Hospital Compare database.

In this paper, we attempt to answer the question, “What types of hospitals are more likely to offer high quality treatment? Are they, for example, the large, the profitable, or the efficient?” Indeed, we find that hospital size, capital structure, asset liquidity, operating efficiency, labor costs, charity care expenses, ownership, and location appear to be important factors of patient care quality. It should not be surprising to learn that the service quality in public hospitals is generally low. Horwitz et al., 2005 [61] argues that public hospitals are more likely to offer relatively unprofitable services than their nonprofit and for-profit counterparts because non-public hospitals are more likely to manage their case-mix carefully and decide which services to offer based on their profitability than their counterparts. Similarly in [62], the authors attribute this phenomenon to the fact that Medicaid patients and patients with severe conditions tend to visit public hospitals.

The results of the first-difference regression suggest that when a hospital generates more profits and takes on more debt financing, its quality of care will generally improve. The time-series effect of profitability on quality supports the theory that hospitals are rational in their choice of service quality when they can earn additional profits when patients’ marginal valuation of quality increases with price [19,21]. The evidence that the use of debt in the capital structure of the hospital has a positive influence on quality of care is consistent with the notion that nonprofit and public hospitals can take advantage of their borrowing capacity stemming from the benefits of tax-exempt bonds [23]. Because financial viability or bankruptcy risk does not seem to be these hospitals’ main concern, they finance operations and investments in quality-improvement related projects, infrastructure, and facilities through the conduit issuance of municipal securities. With the profits they earned and the capital they borrowed, hospitals can train their workforce, employ more highly skilled nurses, improve quality and safety control, reduce patient waiting time, and upgrade medical equipment. These activities eventually improve patient outcomes from quality improvement in the treatment process and infrastructure [37,63]. Finally, it is interesting to discuss the effect of labor costs on service quality. On the one hand, the greater demand for quality services can encourage hospitals to have a high quality workforce, which incurs significant costs in the form of compensation and benefits [30,31], while on the other hand, employing excessive labor can increase hospital costs that will eventually reduce profits [25,26]. The positive relationship we find in the present paper may suggest that, in general, the benefit of hiring more skilled nursing staffs exceeds the cost of the additional wages.

Our study has some limitations that must be considered when interpreting the results. First, the quality score of the Hospital Compare dataset is more an indication of hospital performance on certain processes of care (e.g., for standards and compliance) rather than a measure of treatment outcome (e.g., mortality rate), and the relationship between these two measures is still an ongoing research topic (e.g., [64]). Second, the focus of our study is on the measures of care quality for clinical conditions related to cardiovascular disease and these conditions account for a rather small proportion of hospital admissions; however, we do believe that having a narrowed focus on a small set of medical treatments can ensure a high level of internal validity. Third, our data on hospital financial condition do not provide important details of managerial strategies and incentives that can potentially improve quality measures of treatment processes. Of course, to answer this question would involve the massive and difficult task of interviewing hospital executives and collecting their internal operational data. We will leave such issues for future research.

## Conclusions

Little evidence exists in the literature that addresses the ultimate question of whether hospitals with better financial health are more likely to engage in quality improvement. This paper finds that hospital profitability, financial leverage, asset liquidity, operating efficiency, and costs are important determinants of health care quality. Specifically, the first-difference regression results indicate that the quality rises in the year following an increase in hospital profitability, financial leverage, and labor costs in the same hospital. In addition, public hospitals provide lower quality care than their nonprofit counterparts, and urban hospitals report better quality score than those located in rural areas.

We recognize the potential selection bias of restricting our analysis to only Medicare-certified facilities that filed cost reports as required for receiving reimbursement from the federal government for treating Medicare patients. To make this situation even worse, some hospitals were reluctant to file cost reports on a yearly basis and others reported incomplete information on financial statements and quality measures, and we had to drop observations with missing data from the sample. It should be noted that, although the hospitals in our sample are clearly not representative of all hospitals, they do include several of the most widely recognized and influential medical centers in the United States.

Nonetheless, the results of our study have profound policy implications in this very special sector. While the pursuit of profit induces hospitals to enhance both quantity and quality of services they offer, the lack of financial strength may result in a lower standard of health care services, suggesting the importance of monitoring the quality of care among those hospitals with poor financial health.
